# To Editor of Journal Record of Medicine

**Published:** 1914-03

**Authors:** Dunbar Roy


### TO EDITOR OF JOURNAL RECORD OF MEDICINE.

Allow me space in your Journal to correct an impression which may have been derived from my remarks in presenting a clinical case at a recent meeting of the Fulton County Medical Society. The case in question was in regard to an eye which had been removed on account of injury from a bird shot. In my remarks I was made to cast some uncertainty as to the x-Ray picture made for me by Dr. Derr. I may possibly not have expressed myself clearly on this point. What I intended to say was that while the x-Ray picture did not aid us very materially, yet the shot was found quite near the point of location, but was so imbedded in the sclera as to make it necessary even to do an enucleation. Other cases of foreign body have all been satisfactorily located in the x-Ray pictures taken by Dr. Derr and very fortunately satisfactorily removed.

Dr. Dunbar Roy.

